# Bringing in the controversy: re-politicizing the de-politicized strategy of ethics committees

**DOI:** 10.1186/2195-7819-9-11

**Published:** 2013-11-11

**Authors:** Lonneke Poort, Tora Holmberg, Malin Ideland

**Affiliations:** VU University Amsterdam, Amsterdam, Netherlands; Educational Sciences at Malmö University, Malmö, Sweden; Institute for Housing and Urban Research, Uppsala University, Uppsala, Sweden

**Keywords:** Ethics committees, Controversies, Animal biotechnology, Bio-objectification

## Abstract

Human/animal relations are potentially controversial and biotechnologically produced animals and animal-like creatures – bio-objects such as transgenics, clones, cybrids and other hybrids – have often created lively political debate since they challenge established social and moral norms. Ethical issues regarding the human/animal relations in biotechnological developments have at times been widely debated in many European countries and beyond. However, the general trend is a move away from parliamentary and public debate towards institutionalized ethics and technified expert panels. We explore by using the conceptual lens of bio-objectification what effects such a move can be said to have.

In the bio-objectification process, unstable bio-object becomes stabilized and receives a single “bio-identity” by closing the debate. However, we argue that there are other possible routes bio-objectification processes can take, routes that allow for more open-ended cases. By comparing our observations and analyses of deliberations in three different European countries we will explore how the bio-objectification process works in the context of animal ethics committees. From this comparison we found an interesting common feature: When animal biotechnology is discussed in the ethics committees, technical and pragmatic matters are often foregrounded. We noticed that there is a common silence around ethics and a striking consensus culture. The present paper, seeks to understand how the bio-objectification process works so as to silence complexity through consensus as well as to discuss how the ethical issues involved in animal biotechnology could become re-politicized, and thereby made more pluralistic, through an “ethos of controversies”.

## Introduction

Human/animal relations are potentially controversial and biotechnologically produced animals and animal-like creatures – bio-objects such as transgenics, clones, cybrids and other hybrids – have, when they first appear, often created lively political debate since they challenge established social norms (Franklin 2007; 1997). But animal experimentation is also in itself dilemmatic (Birke et al. 2007), dealing closely with the ideology of “human exceptionalism” (Haraway 2007). The overarching dilemma is expressed in how humans use other animals in biomedical and other research to improve the conditions of our own species, on the one hand, and in how we create and employ various forms of governance to control the conditions and limit the suffering of laboratory animals, on the other. Critical scholars have pointed out how the practices of governance and talk about animal welfare can be viewed as yet another excuse or tool for the machinery to work smoothly (Twine 2010). Moreover, each and everyone involved in animal biotechnology must deal with the “experimental paradox” – simultaneously constructing animals as similar and dissimilar to humans^a^. Ethical issues regarding the animal/human divide, the boundaries between living/non-living and the matter of animal welfare have at times been widely debated in many European countries and beyond (Harvey & Salter 2012; Urbanik 2007). However, the general trend is a move away from parliamentary and public debate towards institutionalized ethics and technified expert panels, at both the national and EU level. But what does it mean that these complex issues have become reduced to ethical terms that are handled by only a few? In the present paper, we explore what effects such a move can be said to have, and counter by suggesting a revitalized political debate.

One common feature of novel, bio-medically produced forms of life – bio-objects – is that they are potentially controversial because they challenge societal, institutional, legal and cultural matrixes (Vermeulen et al. 2012). At a policy level, these quite luxuriant entities are often handled and regulated by way of reduction, thus reducing heterogeneity to homogeneity through re-framing, re-categorization and boundary work. One example is the British cybrid debate, in which the species identity controversy was taken up in the parliamentary debate through numerical evidence, thus framing the cybrids as more animal than human and consequently as entities that should be handled by the animal side of the institutional and legal apparatus (Brown 2009a; Haddow et al. 2010). This is an example of a bio-objectification process, in which an unstable bio-object becomes stabilized and receives a single “bio-identity” (Holmberg et al. 2011) by closing the debate. However, we argue that there are other possible routes bio-objectification processes can take, routes that allow for more open-ended cases.

Using this conceptual lens of bio-objectification, we compare different studies to explore how the bio-objectification process works in the context of animal ethics committees. By comparing our observations and analyses of deliberations in three different European countries, we can separate out an interesting common feature: When animal biotechnology is discussed in the ethics committees, technical and pragmatic matters are often foregrounded. At the same time, complex issues are downplayed. Thus, despite the differences in institutional and legal frames, there is a common silence around ethics and a striking consensus culture (Moreno 1995). By this we mean that a potential plurality of viewpoints is fostered into a singular “discourse”. This discourse or way of speaking about dilemmas in animal biotechnology dominates by setting the frames for discussion.

Restricting to a singular “discourse” in the bio-objectification process brings in the advantage that it becomes more likely that a consensus will be reached. In all three countries we have noticed a strong consensus culture. In deliberative democracy theories, two conceptions of consensus can be distinguished (Poort 2013). First of all, reaching for consensus can structure debate by bringing parties together focusing on similar aims. Consensus is a dynamic concept in which consensus is a regulative ideal. Consensus is, then, an orienting aim while the outcome is less foundational (Gutmann et al. 1996). Second, consensus is considered the most ideal outcome of a rational debate. Consensus in this sense is a more static concept. Consensus as the ideal outcome depends on the ideal political process in which is aimed for consensus under the ideal circumstances being given by the rationality of debate (Habermas 1989).

Understanding the bio-objectification process as a deliberative process, consensus seems to be considered as a more dynamic concept in which a new unstable bio-object is explored and discussed. At the same time, it is aimed to stabilize the bio-object and develop a new bio-identity. Here, we can notice an interplay between consensus as a regulative ideal and consensus as an ideal outcome by which the rationality of debate is emphasised.

The present paper, thus, seeks to understand how the bio-objectification process works so as to silence complexity through consensus as well as to discuss how the ethical issues involved in animal biotechnology could become re-politicized. Thereby it can be made more pluralistic, through an “ethos of controversies” (Poort 2012). The paper is based on empirical studies of ethics committees, as the general trend has been to appoint such committees, resulting in a reduction of complex issues into a kind of “rational” reasoning ^b^. However, the purpose is not to present new empirical data, but to use them to discuss how to bring complexity into animal ethics. However, the discussion may be relevant to a broader spectrum of issues related to bio-objects and bio-objectification.

## Context and methodology

The present article aims to explore the bio-objectification process and the role of animal ethics committees by drawing on two independent studies. The first study compared the regulation of animal biotechnology in the Netherlands and Switzerland to illustrate the use of alternative legislative strategies when addressing complex issues^c^. The second study is a part of the project *Dilemmas with transgenic animals*, which focused how dilemmas associated with transgenic animals were (not) handled in laboratories and animal ethics committees in Sweden (Ideland 2009; Holmberg & Ideland 2009; Holmberg & Ideland 2012a; Holmberg & Ideland 2012b).

### Context

Both the Netherlands and Switzerland have regulated a distinct licensing procedure for animal biotechnology in addition to licensing of animal experiments. It was argued that animal biotechnology required a separate regulation, because animal biotechnology brings with it distinct controversies and novel challenges to the moral status of animals. In that context, this comparative study analysed the role of the Dutch Ethics Committee on Animal Biotechnology (CAB)^d^ and the role of the Swiss Ethics Committee on Non Human Gene Technology (ECNH). The CAB is a committee of nine experts from various fields (social science, biotechnology, medical science, and ethics). The ECNH is a committee consisting of about twelve members from different fields of expertise (biotechnology, social science, medical science, ethics, and law). No lay people are involved in either of the committees.

Both committees were officially assigned to advise on cost-benefit evaluations in concrete cases as well as to give advice on the moral impact of issues concerning non-human gene technology. In both countries, it was eventually the Minister who was responsible for licensing.

In the Netherlands, the CAB had a prominent role in licensing. During the licensing procedure, a scientist submits an application to the Minister of Agriculture, who sends it to the CAB. The CAB formulates its recommendation based on an ethical review. After receiving this recommendation, the Minister draws up a draft decision. This draft decision is opened to public objections. The public can then explain their objections in a public consultation procedure organized by the Minister. In reality, it is the CAB that chairs these hearings. After considering the public comments, the CAB advises the Minister again. Officially, the Minister then decides whether or not to grant the license. In the licensing procedure, however, the Minister almost always adopts the advice of the CAB. Thus, in reality, it is the CAB that plays the decisive role. The Minister follows an ‘no, unless’ policy which entails a restrictive use of animals in biotechnological procedure. However, in reality, 90 per cent of all applications were based on a consensus. The CAB, and therefore the Minister, seem to have adopted a ‘yes, if’ policy (Meijer et al. 2005; Paula 2008).

In Switzerland, the Biotechnology Section of the Federal Office for the Environment is the authority responsible for licensing. Most prominent and important in the licensing procedure is the consultation of the two expert committees. The consultation process starts with the Swiss Federal Expert Committee for Biosafety (SECB), which advises the Biotechnology Section concerning biosafety risks. After the biosafety consultation, the ECNH is consulted on moral matters, taking into account the SCEB’s advice. The ECNH is not obliged to give advice on each license application, and therefore restricts itself to controversial or new issues raised by particular applications. As a result, the ECNH can respond to new trends and think through the consequences and moral impact of these trends. In effect, this means that a moral evaluation is only performed if the ECNH classifies an application as morally controversial. Consequently, if the ECNH refrains from giving advice, the Biotechnology Section can presume that the license application is not controversial and does not require further moral deliberation (Errass 2006).

In Sweden, seven local animal ethics committees examine applications for all non-human animal experiments. The body responsible for the apparatus is the Ministry of Agriculture. The ethical evaluation is mandatory for all projects including animal experimentation, and the Swedish committees play a decisive role. The ethical evaluation is organized such that the responsible researcher sends in an application for the experiment, which includes a description of experimental procedures, anaesthetics to be used, the ‘endpoint’ of the experiment, and the degree of suffering that could be expected. Concerning transgenic animals, it is business as usual, with one exception; you have to state in the application that the project concerns genetically modified animals (Nordgren & Röcklinsberg 2005).

Each of the seven committees consists of a chairperson, six scientific experts and six laypersons. The scientific experts come from universities and pharmaceutical companies; this category also includes animal technicians and veterinarians. The laypersons represent local political parties, animal welfare and animal rights organizations. In the Swedish case, the committees’ task is to make cost-benefit evaluations: Is the animals’ suffering counterbalanced by the expected benefits of the research in a specific experiment? They are also supposed to discuss every application on the basis of The Three Rs: Refinement, Reduction and Replacement (Russel & Burch 1959). Despite the heterogeneous composition of the Swedish committees, 99 per cent of applications are approved, sometimes with requested modifications.

In other words, there are differences between the three countries in how the ethical evaluations and the licensing are carried out. In the present article, we do not intend to discuss how these different contexts shape different conditions for the ethical reviews. Instead we focus on the similarities in the outcomes, which we find remarkable.

### Methodology

The studies used different approaches as they were set up within different disciplines, therefore different data collection methodologies were used as well. In the Swedish study, twenty committee members, representing the different categories of members, were interviewed during 2006 and 2007. The interviews were semi-structured, participant driven and lasted on average 1.2 hours. In addition, twelve meetings in six different committees were observed^e^. In the comparative Dutch and Swiss study, data were collected by analysing the committees’ annual reports, including their advices, the internal and external evaluations of the committees’ works, along with interviews with members and involved actors^f^, parliamentary explanatory documents, and interviews with committee members, responsible institutions, and key figures in licensing.

## Depoliticizing animal biotechnology

From the interview study on Swedish animal ethics committees, we see that the individual interpretations of the evaluation task differed^g^. Basic researchers without specific clinical interests meant that their task in the committee was to empower scientific research and hinder “ animal activists” from stopping it: *“We have to be there to make it easier for science. If we don’t work actively with these questions, they never get the right input from us.” (Scientific expert).* Other committee members, both clinical researchers and lay people, stated that the point of the ethical evaluation was to help future patients: *“…for the individual patient, families, society. Everything” (Scientific expert).* Further, some of the members stated that the main aim of the evaluation was to protect the wellbeing of experimental animals. This view was represented in interviews with researchers as well as with lay people and representatives for animal welfare or animal rights organizations. However, the people from animal rights organizations expressed a feeling of being kept hostage in the committee, as their political agenda was more or less impossible to raise without the risk of being categorized as a fundamentalist activist – not worthy to listen to: *“Principally I am against all animal experimentation, no matter how they are done, and use of animals for our purposes. So what I do in the committee is just fixing the surface and improving the animal’s existence” (Lay person for Animal rights)*.

Animal rights representatives acquired the strategy to refer to scientific journals on how to decrease animal suffering or replace animal experimentation with other kinds of research methods instead of claiming animal rights. Lay people from political parties were, on the other hand using a strategy of silence during the meeting. These both strategies reflect how the Swedish committee culture is characterized by a strong scientific discourse. Inside this, animal rights representatives feared to be constructed as fundamentalists and political lay people were afraid of appearing as “stupid”, since the issues were “difficult” from a scientific and technological point of view: *“Some scientists are snotty. They think that we are asking silly questions and that we should be kind to them” (Political elected lay person).*

The intersection of different views on the aim of ethical evaluation could be an interesting foundation for pluralistic ethical reasoning. But inside the hegemonic scientific discourse and the downplaying of other perspectives, the discussions focused almost exclusively on technical and methodological improvements of the experiments. Complex ethical questions about research purposes and animal suffering in this context became unspoken (Forsman 1993), even though members agreed that they had been appointed to conduct the evaluation.

Beside the strong scientific discourse, another interpretation of why mainly technical problems were discussed is that, on these issues, committee members could reach a consensus. Due to their differing perspectives, it was difficult – or perhaps even impossible – for the members to reach a consensus on whether the research purpose was good enough to justify animal suffering. But one theme all members could agree on was how to decrease the suffering of animals with better technical solutions, which is also reflected in the requested technical modifications of the experiment that was often communicated from the committee to the researcher. Solutions could be found in relation to the more technical aspects, at least they could agree on a humane end-point, when the animal should be put to death. Better technical solutions seemed to benefit scientific research as well as the animals. They probably also benefit patients. Decreasing suffering can be done in the name of science, in the name of patients and in the name of animals – and therefore it was also a relatively easy goal for a discussion aimed at reaching consensus. The interplay between consensus as the ideal outcome and consensus as a regulative ideal seem to be overruled by the first. Within the ethics committees, the need for defining the bio-identity is emphasised. The process of exploration of the bio-object in which all controversies are discussed, is consequently downplayed.

The Swiss and Dutch ethics committees had been formally assigned an advisory role, and were supposed to make cost-benefit evaluations as well as to crystallize general norms on the moral status of animals. However, in both countries, it was ultimately the Minister who was responsible for licensing.

In the Netherlands, the CAB had a prominent role in licensing. Officially, the Minister of Agriculture, Nature and Food Quality then decides whether or not to grant the license. In the licensing procedure, however, the Minister almost always adheres to the advice of the CAB. Thus, in reality, it is the CAB that plays the decisive role. This role changes the discourse for the CAB and limits the deliberations on individual cases and did not reach a more fundamental level. Paula has identified three different strategies that the CAB uses to develop decision-making norms^h^. First, the CAB has developed substantial consensus norms. Second, the CAB has developed various procedural norms for dealing with a lack of data or criteria. These procedural norms can postpone a substantive judgement. Third, the CAB has developed a strategy of reframing the issue^i^. All these strategies are intended to smooth over moral conflicts.

Concerning the first, these norms were merely limited to developing standards for more practical and technical matters. Developing standards also inclined the CAB towards the third strategy: reframing the issue. The issue was reduced to more practical and technical matters. Regarding these matters, the CAB was able to come to a unanimous decision in 90 per cent of the cases. It seems that processes of fact-finding and constructing norms contributed to the development of a set of well-functioning practical standards and concrete norms. However, the CAB has done little to develop the moral status of animals, as the issue was no longer discussed in the committees’ discourse. Bovenkerk questions whether the 90 per cent rate of unanimous decisions reflects true consensus or whether this ‘means that the more divisive moral problems were excluded from Committee discussions?’ (Bovenkerk 2011). Again, the need to define the status of animals in a political context overrules the process of exploring all controversy involved with the new and unstable bio-object.

It is noteworthy to point out that there was deliberation on the moral issues in the licensing procedure. However, deliberation became what can be called a ‘ritual dance’^j^, referring to the repetitive nature of the interactions between the CAB and commentators on licensing applications. During consultation rounds, citizens and stakeholders are only meant to submit views, while objections or a real debate are empathically discouraged, giving rise to frustration among the participants. The submissions are reviewed by the Committee, but seldom influence its final decisions. This is because the same small group of stakeholders—mainly from animal protection and alternative medicine organizations—tend to submit the same objections over and over again and these objections are, therefore, already anticipated by the Committee in its recommendations. This means that in its draft recommendations any possible objections are already mentioned, but often dismissed out of hand by simply stating that the majority of the Committee disagrees. The Chair’s answer in a public hearing to a question posed by a representative of the animal protection organization, on whether alternative approaches should be considered, exemplifies this tendency. The Chair answered by stating that ‘*this is not the right forum to discuss the alternatives*’ (Bovenkerk & Poort 2008). This process has resulted in a standardization of both the stakeholders’ objections and responses to these objections by the CAB.

In contrast to the Dutch committee, the ECNH is not limited to a legalistic context, as the ECNH is not obliged to give advice on each license application. As a result, the ECNH can respond to new trends and can think through the consequences and moral impact of these trends. Consequently, their deliberation can really be positioned in the moral discourse. Ethical review merely focused on concrete cases based on a cost-benefit analysis in terms of animal suffering. Nevertheless, there was room for more general debate on the dignity of living beings. However, a further crystallization of the moral status of animals was limited^k^.

This procedure forecloses the debate on controversial positions that are not represented in the Committee. The ethical review of the ECNH was pre-structured by an interpretation of dignity of living beings as a gradual concept introduced by the ECNH, which was in itself a controversial concept. The concept of dignity of living beings was not build on a consensus among its members and was still highly controversial. During the legislative process it was emphasized by the ECNH that the concept required further fundamental debate. Besides advising on licensing, the ECNH was assigned to engage in such a debate. At the same time, the ECNH’s role in licensing could justify the exclusion of public consultation in licensing and in further deliberation on the status of animals. Consequently, dissenting concerns and viewpoints were not re-introduced in licensing practice and moral deliberation was limited to the ECNH’s members who launched the gradual concept. This concept started to function as if it were based on a consensus, whereas it was actually a temporary deficit and resulted in the early stagnation of norm development concerning the dignity of living beings. The strong drive for consensus precluded attempts to alter the definition and the essence of the ‘dignity of living beings’.

A good example is the ECNH’s statement about a license application for a field trial with primates by Zürich’s ETH in 2007^l^. Both the ECNH’s own discussion and its response reflected fundamental disagreements on interpretation of the definition of ‘dignity of living beings’. It was not at all clear whether the interpretation of 'dignity of living beings’ as a gradual concept was justified or how it could be explained in the individual case. This shows that the ECNH’s interpretation was not based on consensus among the parties: instead, the parties were diametrically opposed^m^. The strong drive for consensus resulted in constructing ‘dignity of living beings’ as a gradual concept, as if it were based on a consensus. In reality, this consensus was superficial, and many divergences still existed. The search for consensus prematurely stifled further exploration of the concept, and undermined reflection on the concept’s moral meaning^n^.

Although the ethics committees in all the studies operated in different contexts, their roles and aims are comparable. They were assigned to review licensing from an ethical perspective. In Switzerland and the Netherlands, they were even asked to define the moral status of animals in light of animal biotechnology in general. Despite their task, the ethical issues were not the main focus; instead the committees merely standardized practical and technical matters. In all case studies, we identified influences of a strong consensus culture. This has at least two consequences that were visible in the outcomes of these case studies. First of all, deliberation was limited to a level on which consensus could actually be reached: the more practical and technical matters. Second, debate was dominated by a single perspective and was foreclosed to controversial or dissenting viewpoints. In Sweden and Switzerland, there was no room for the public to express their viewpoints and concerns. Ethical review in itself was considered sufficient. In the Netherlands, there was room for public objection. However, these objections were easily downplayed ending in a ‘ritual dance’. Consequently, dissenting viewpoints were not taken seriously. Using the conceptual lens of bio-objectification here, we can conclude that bio-objectification is reduced to homogeneity in which the singular perspective of techno-science dominates. The complexity of the issue is silenced, which leads to a foreclosure of debate and of the bio-objectification process. Bio-objectification as a process of exploring the unstable bio-object became a process in which the ‘new’ bio-object was ‘identified’ as soon as possible. Although identification is part of the process of bio-objectification, the tendency within these practices of ethics committees seems to be that identification becomes most important. Our analysis is, that the interplay between exploration and identification (consensus as dynamic concept ad consensus as a static concept) is dominated by identification. As a result, complexity and controversy are silenced. We question whether bio-identification may be too premature risking depoliticizing animal biotechnology and simplifying the debate on the status of animals.

## Re-politicizing animal biotechnology

The previous section has pinpointed the side effects of depoliticizing animal biotechnology by reducing bio-objectification to institutionalized ethics committees. As we have noticed, despite the differences in legal and institutional framing, the discourse of the ethics committees in the three countries can be identified as *deliberative* discourses. Deliberative democracies promote an institution of deliberation procedures that meet the standards of rationality, consensus and democratic legitimacy. We have discussed two concepts of consensus by which these standards can be met. Habermas argues for a communicative rationality, which he means will occur when the right conditions are met in an ideal speech situation, e.g. when citizens are equal and free to express their viewpoints. In this ideal speech situation, the ideal outcome is the establishment of a rational consensus^o^. A second concept involved consensus as a more dynamic concept in which consensus is not necessarily the ideal outcome, but functions as an orienting aim. The bio-objectification seemed to illustrate an interplay of both conceptions. Exploration of the bio-object can be seen as an orienting aim, while identification can be considered as the ideal outcome. The interplay between both should ensure that the identification reflects the establishment of a rational consensus in which all viewpoints and difficulties are deliberated.

However, the case studies prove otherwise. In their striving for consensus, the diverse perspectives were downplayed. The frame for discussion was limited to a singular dominant discourse in which the potential plurality of perspectives was foreclosed. In the following, we will discuss how ethical evaluations can be made from a more pluralistic perspective by changing from a consensus culture to a culture of controversy.

Mouffe criticizes the rationality of the deliberative discourse (Mouffe 1999). To her, one crucial shortcoming of the deliberative approach relates to its distinction between the private and public sphere, where the public sphere is seen as a neutral domain in which rationality is at the forefront, while the private sphere is seen as a domain in which a plurality of viewpoints on what constitutes ‘the good life’ can exist. Mouffe argues against this strict distinction from the perspective that the public and the private domain intermingle. People do build their democratic values based, among other things, on passions and emotions; therefore the public sphere cannot refrain from personal interests and particularities. Consequently, searching for consensus always tends towards exclusion of interests; they are excluded from what is seen as “rational” in the ideal deliberative democracy. Examples from our case studies are how animal rights spokespersons were constructed as radical fundamentalists and lay people as “not knowing”. Both were in that sense excluded from “rational” decision-making.

Another aspect of Mouffe’s fierce critique of the deliberative rationality is the fact that she recognizes acts of power in every speech act. She is not interested in overcoming or neutralizing power struggles by means of debate. Instead, Mouffe argues, the aim of democracy is to constitute forms of power that are more compatible with values of democracy^p^. This aim involves a radical change of the conception of the we/they opposition. Mouffe suggests an ‘agonistic pluralism’ in which ‘they’ is no longer considered as an enemy to be excluded, but as an adversary, a legitimate opponent who is defending a different opinion. Different perspectives must then be recognized rather than silenced.

So, with help from Mouffe, we argue that the debate, and thus the bio-objectification process, be opened up for pluralistic viewpoints that can re-politicize the bio-objectification process. We mean that there is both a need to brings ethics into the ethics committees and a need to bring the issue out into more public contexts. However, the latter solution is not as easy as it seems. Harvey and Salter^q^ have pointed out that public engagement in ethical issues per se would not involve a pluralistic discussion. Instead, it might contribute to the stabilization of consensus. Our suggestion is that this is done by establishing an ethos of controversies in ethics committees, and other contexts for discussing and handling complex issues on biotechnology^r^. An ethos of controversies is a normative way of thinking in which controversies are the main focus in decision-making and politics. This ethos structures decision-making processes and norm development around an exploration of controversies. In the light of agonistic pluralism, this new ethos does not claim to offer an alternative for exclusion of perspectives; neither does it claim to ensure inclusion of all conflicting viewpoints. The ethos of controversies is, however, not a method of decision-making. We do not claim that an ethos of controversies presents an alternative to decision-making or to identification^s^. We realise that decisions have to be made as well as bio-objects need to be identified. Controversy cannot justify these decisions or the identifications of bio-objects. Instead, an ethos of controversies restructures political processes. An ethos of controversies is built on the need to acknowledge the political nature of decisions and to consider decisions as temporary phenomena in an on-going confrontation. Opening the seemingly closed bio-objectification process in the ethics committees, and thereby de-stabilizing the bio-identity, may do justice to the complexity of the issue of animal biotechnology. In this way, the interplay between exploration of the bio-object and the identification of the bio-object in the bio-objectification process can be re-introduced.

## Final remarks

The case studies have shown that there is a tendency to depoliticize complex issues by reframing heterogenic standpoints and placing them in a homogenic discourse, thus silencing the ethical dilemmas involved. Moreover, institutionalizing and compartmentalizing the dilemmas associated with animal experimentation will in turn conserve science/public relations^t^. Studies on animal research and animal experiments have shown that it is not only in the context of institutional policy- and decision-making that (controversial) ethical considerations are silenced. In the public debate, there is a similar tendency to restrict debate to applications and scientific matters, silencing the opposition either through co-option, or “marginalisation through inclusion”^u^, or by classifying it as irrational and unscientific (Druglitrø 2012; Asdal 2008). The controversial opponents to animal research are outlawed by referring to them as irrational ‘activists’, which deprives them of a legitimate position in public debate as well (Brown 2009b). Instead, the public debate tends to reduce debate to a welfare discourse, and by doing so polarizes the available positions as either rational or irrational.

Reducing dilemmas to a singular discourse risks foreclosing the debate and the bio-objectification process. We therefore argue for a re-politicization of animal biotechnology by opening up the debate to pluralistic viewpoints and perspectives through the introduction of an “ethos of controversies”. In this way, the process of bio-objectification can be widened. According to Mouffe, channels are needed through which the pluralistic viewpoints can be expressed. However, we do not argue for another tool to bring to the “public”. Existing tools for public engagement such as public trials, consensus conferences, and focus groups have already been established and evaluated/criticized by STS scholars (Irwin 2006; Kelly 2003). Instead, we propose that what is needed is for the various discourses on law, policy, science, and ethics to be moved from one single institution – ethics committees – to a plurality of arenas that at times can intermingle, as a means to acknowledge the power asymmetries now built into the decision-making process.

This requires both additional channels and restructuring of old ones. An ethos of controversies aims at a radical change in the culture of deliberation within these channels to create space for controversy. Instead of emphasizing the rationality of the debate, we argue for the need to acknowledge the political nature of decision-making, which always involves exclusion. Moreover, we suggest refraining from goal-setting in an early stage in terms of laws, consensus, or specific kinds of policy for which debate is a means. Instead, we propose stimulating ongoing debate as an end in itself, and thus, bio-objectification processes as ongoing confrontations in which law and policy are only a temporary and political respite. Law-making and policy-making are, thus, part of the broader circular process that feeds the political debate, while the debate in turn feeds into politics. By acknowledging the plurality of discourses together with the political nature of ethical decisions, law-making and policy-making, opponents can contribute to the debate through passionate politics and channels other than institutionalized ethics committees.

## Endnotes

^a^Birke, Arluke & Michael, cite 2.

^b^Harvey and Salter, cite 6.

^c^Poort, cite 11: chapter 6 and 7.

^d^Recent developments are not included in this research, as the CAB no longer operates in legislative practice. This study concerns the function of the Decree until July 2009. Remarkably, the Dutch Minister decided in July 2009 that the CAB would no longer be required for exploring the moral status of animals in light of animal biotechnology. According to the Minister, the number of unanimous decisions made clear that the moral status had been crystallized. The members of the CAB questioned this conclusion. The chairman stated that even though they have reached unanimous decisions and substantial consensus on several biotechnological procedures with animals, the moral status requires further debate.

^e^Ideland, cite 15: 258–261.

^f^Paula, cite 17 Meijer, cite 17.; Erras, cite. 18.

^g^Ideland, cite 15: 258–261.

^h^Paula, cite. 19: Chapter 3.

^i^Paula, cite 19: 78–80.

^j^Paula, cite 19: 3.

^k^It must be mentioned that the Swiss Ethics Committee on Non-Human Gene-technology (ECNH) has published a few statements presenting a more general reflection on the dignity of living beings. Furthermore, associated academics have contributed to defining and exploring the concept. However, these explorations have mostly been confined to an academic debate on the extent of the gradual concept as it applies in particular cases, and have not addressed the fundamental substance of the concept itself.

^l^Interview with A. Willemsen, March 2007.

^m^Some of the members of ECNH partly disagree, but nevertheless, the statements of the ECNH are presented to the public and the authorities as a unanimous decision, as has became clear during personal communication with members of the ECNH, 2007. J. Fischer, ‘Haben Affen Würde?’ (ethik.uzh.ch July 2007) <http://www.ethik.uzh.ch/ethikkommission/veranstaltungen2/Downloads/EK-JF-Haben-Affen-Wuerde.pdf>, accessed July 2007.

^n^Bovenkerk and Poort, cite 31: 27.

^o^Habermas, cite 13.

^p^Idem.

^q^Harvey and Salter, cite 6:193–199.

^r^Poort, cite 11.

^s^Poort, cite 11: 165.

^t^Holmberg and Ideland, cite 17: 354–368.

^u^Harvey and Salter, cite 6: 193–199.
